# Dilemmas in the Management of Digital Ulcers in Systemic Sclerosis–Mixed Connective Tissue Disease: Lessons From a Case Report

**DOI:** 10.7759/cureus.93477

**Published:** 2025-09-29

**Authors:** Khushali Dadhich, Miet Shah, Ashish Jain, Niharika Gill

**Affiliations:** 1 Medicine, K. J. Somaiya Hospital and Research Centre, Mumbai, IND; 2 General Surgery, Bedfordshire Hospitals NHS Foundation Trust, Luton, GBR

**Keywords:** amputation, digital ulcers, lower-limb ulcers, mixed connective tissue disease, multidisciplinary care, raynaud’s phenomenon, systemic sclerosis, vasculopathy

## Abstract

Systemic sclerosis (SSc) is a rare autoimmune disorder marked by fibrosis and microvascular injury, commonly manifesting as digital ulcers and skin thickening. Lower limb ulcers are uncommon but can contribute to significant morbidity, need prolonged healing, and carry the risk of progression to necrosis or amputation, particularly in systemic sclerosis-mixed connective tissue disease (SSc-MCTD). We report a case of a 57-year-old woman with Raynaud’s phenomenon, inflammatory small-joint arthritis, alopecia, photosensitive rash, and exertional dyspnea. Despite improvement in arthritis with disease-modifying antirheumatic therapy, a lower limb ulcer progressed while she was on vasoactive therapy (α-adrenergic blockade, phosphodiesterase-5 inhibition, endothelin-receptor antagonism) and immunomodulation, culminating in necrosis and pain that required amputation of the great toe and subsequently the forefoot. Laboratory evaluation revealed systemic inflammation and dyslipidemia, while vascular imaging demonstrated both microvascular and macrovascular compromise. Histopathology confirmed acute inflammatory changes without primary vasculitis. Rheumatology evaluation and serology established a diagnosis of SSc-MCTD, after which management was refocused on targeted vasodilation, immunomodulatory therapy, and multidisciplinary wound care. This case underscores the central role of vasculopathy in SSc-MCTD, highlights the limited efficacy of surgical intervention without optimized medical management, and emphasizes the importance of early involvement of Rheumatology and coordinated multidisciplinary care to prevent progression to irreversible ischemic injury.

## Introduction

Systemic sclerosis (SSc), also known as scleroderma, is a rare and heterogeneous autoimmune connective tissue disorder characterized by progressive fibrosis of the skin and internal organs. Its global prevalence is estimated at approximately 200-250 cases per million, with a marked female predominance [1]. The underlying pathology is defined by a triad of small-artery intimal proliferation, medial thinning, and adventitial scarring [2], resulting in widespread vasculopathy with subsequent cutaneous and visceral fibrosis [3]. Among the earliest clinical manifestations are Raynaud’s phenomenon and digital ulceration, which contribute significantly to morbidity, disability, and impaired limb function. Digital ulcers are reported in more than half of patients during the disease course and typically arise as a consequence of advanced vasculopathy [4].

Systemic sclerosis is currently classified according to the 2013 American College of Rheumatology/European League Against Rheumatism (ACR/EULAR) criteria, which provide greater sensitivity for early disease detection and integrate both clinical and serological domains [5]. Clinically, the disease is still recognized as diffuse cutaneous and limited cutaneous subtypes, with Raynaud’s phenomenon typically preceding other systemic manifestations by several years in the limited form, but coinciding with or shortly preceding internal organ involvement in the diffuse form [6]. Beyond these classical subsets, systemic sclerosis-mixed connective tissue disease (SSc-MCTD) has been recognized as a distinct phenotype, defined by SSc features in conjunction with high-titer anti-U1-RNP antibodies and additional manifestations of other connective tissue disorders. This phenotype demonstrates characteristic patterns of organ involvement and prognosis that distinguish it from isolated systemic sclerosis [7,8].

Lower limb ulcers occur in approximately 12%-13% of patients with systemic sclerosis, a prevalence much lower than digital ulcers but associated with greater morbidity. Healing is typically protracted, and these ulcers carry a substantial risk of recurrence and limb loss, particularly when macrovascular disease coexists [9]. The management of ischemic ulcers in SSc and SSc-MCTD is challenging; while most respond to vasoactive and immunomodulatory therapy, progression to severe tissue loss is uncommon and surgical intervention is usually considered only for irreversible ischemia or uncontrolled infection [10].

The present case describes an uncommon clinical course in which a lower limb ulcer progressed to gangrene and necrosis requiring amputation, a complication rarely reported in SSc-MCTD, where ischemic involvement typically affects the digits. This report underscores the importance of early recognition of autoimmune vasculopathy, the limitations of pharmacologic therapy in refractory disease, and the crucial role of general surgical intervention when advanced complications culminate in irreversible tissue loss.

## Case presentation

A 57-year-old woman presented with Raynaud’s phenomenon, accompanied by dryness of the mouth, throat, and eyes. She also reported inflammatory arthritis affecting the small joints of the hands, alopecia, and photosensitive rashes. Progressive exertional breathlessness corresponded to New York Heart Association (NYHA) class II, defined as dyspnea with ordinary physical activity, and was noted in association with secondary hypertension, though the precise etiology was not yet established.

She had previously been treated by a local physician for five years with disease-modifying antirheumatic drugs (DMARDs), specifically methotrexate and leflunomide. This regimen led to the resolution of arthritis and improvement in Raynaud’s phenomenon, but during the same period, she developed progressive skin tightening and recurrent ulceration of both upper and lower limbs.

At presentation with worsening ischemia, laboratory investigations were prioritized to evaluate anemia, inflammation, infection risk, and systemic involvement, given the context of a possible connective tissue disorder where both vasculitis and thrombotic non-vasculitic causes of ulcers may occur [11]. The complete blood panel demonstrated anemia, thrombocytopenia, micronutrient deficiencies, mildly impaired renal function, and markedly elevated CRP, confirming a systemic inflammatory state consistent with the clinical picture. These results are summarized in Table 1. Although dyslipidemia was present, the patient lacked traditional cardiovascular risk factors such as diabetes, smoking, or primary hypertension; her only contributory background risks were a sedentary lifestyle and suboptimal diet.

**Table 1:** Laboratory results The patient demonstrated hematological abnormalities (severe anemia and thrombocytopenia), evidence of dyslipidemia, micronutrient deficiencies (vitamin D and iron), mildly impaired renal function, and a markedly elevated CRP indicating significant systemic inflammation. Boldfaced values represent those outside the laboratory reference ranges. Hb: hemoglobin; WBC: white blood cell; Plt: platelet; CRP: C-reactive protein; HDL: high-density lipoprotein; LDL: low-density lipoprotein; TIBC: total iron-binding capacity; HbA1c: glycated hemoglobin

Lab test	Result	Biological reference interval
Hb	6.8 g/dL	12.0-15.0 g/dL
WBC count	7860/µL	4000-10000/µL
Plt count	123 x 10^3^/µL	150.0-400.0 x 10^3^/µL
Total cholesterol	202 mg/dL	<200 mg/dL
HDL cholesterol	22 mg/dL	40-60 mg/dL
LDL cholesterol	128 mg/dL	<100 mg/dL
Triglycerides	159 mg/dL	<150 mg/dL
Serum creatinine	1.17 mg/dL	0.6-1.1 mg/dL
Total bilirubin	0.84 mg/dL	0.3-1.2 mg/dL
Alkaline phosphatase	73.1 U/L	45-129 U/L
Aspartate aminotransferase	23.6 U/L	<35 U/L
Alanine transaminase	24.1 U/L	<45 U/L
25-OH vitamin D	19.44 ng/ml	20.0-40.0 ng/ml
Serum B12	677 pg/ml	197-771 pg/ml
Serum TSH	2.38 mIU/L	0.25-5.0 mIU/L
CRP	81.24 mg/L	<1.00, low risk; 1.00-3.00, average risk; >3.00-10.00, high risk; >10.00, possibly due to non-cardiac inflammation
HbA1c	5.8%	0.0-6.0%
Serum ferritin	50.3 ng/mL	13-150 ng/mL
Serum iron	16 µg/dL	Male: 65-175 µg/dL; female: 50-170 µg/dL
Transferrin saturation	31%	13%-45%
TIBC	192 µg/dL	Male: 225-535 μg/dL; female: 215-535 μg/dL

**Figure 1:**
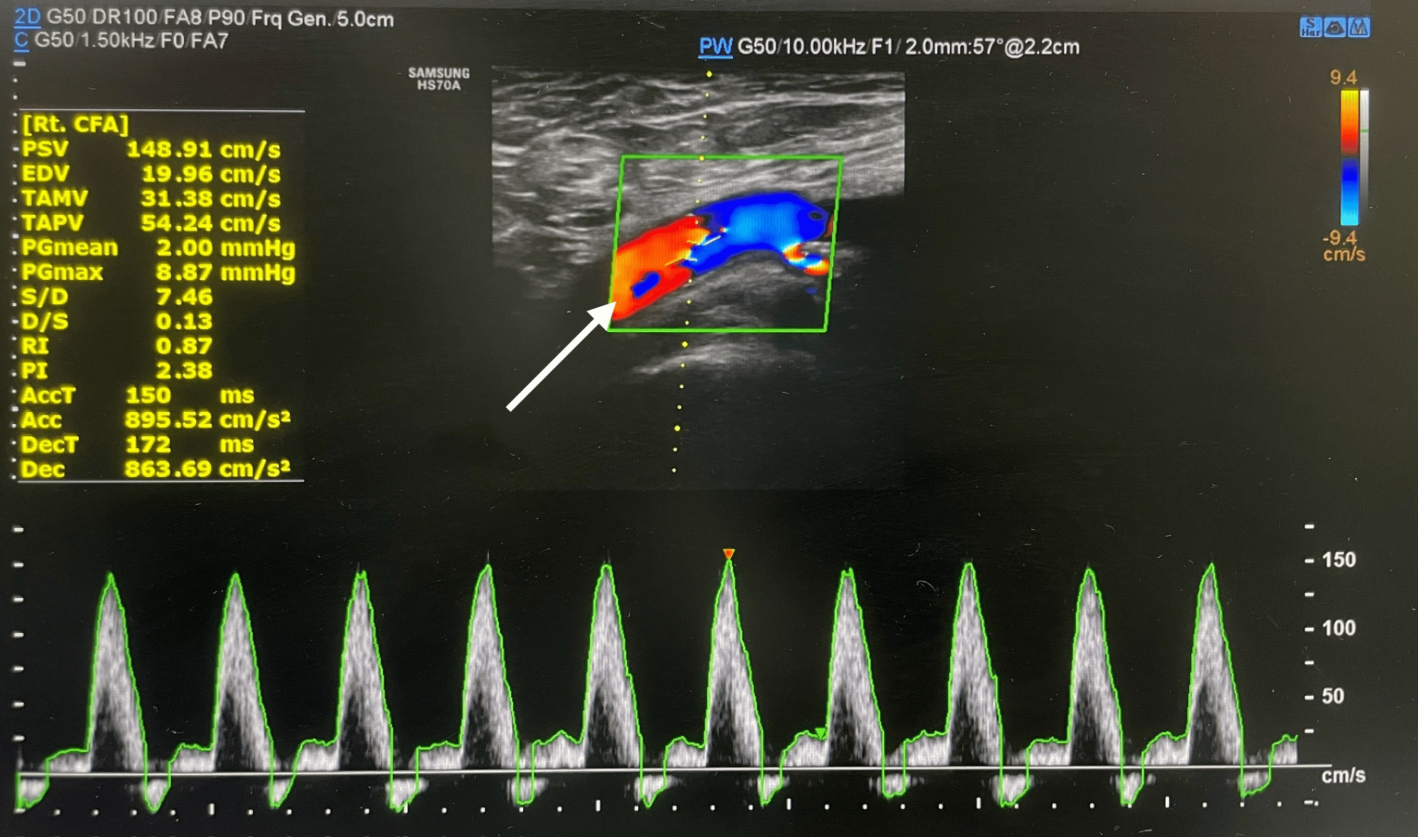
Right lower limb arterial Doppler showing normal flow

**Figure 2:**
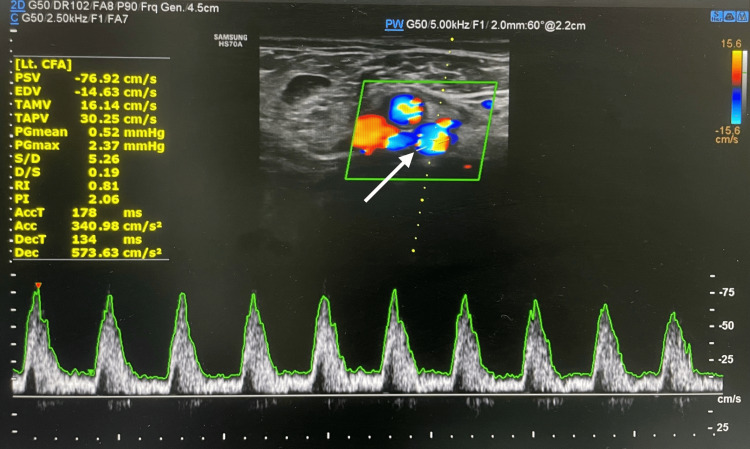
Left lower limb arterial Doppler showing feeble flow with the blue areas showing reduced flow velocity

To further characterize the ischemia and distinguish microvascular disease from superimposed macrovascular pathology, arterial Doppler ultrasound was performed. This demonstrated normal arterial flow on the right (Figure 1) and markedly reduced flow velocity on the left, consistent with peripheral atherosclerotic arterial disease (Figure 2). Identifying large-vessel involvement was important, as ulceration may arise from inflammatory vasculitis or thrombotic mechanisms, and recognition of a proximal stenosis would directly influence surgical decision-making [12].

In the setting of vascular compromise and a contaminated wound bed, the patient was referred to plastic surgery, where sharp debridement and regular dressings were performed. Although a surface swab subsequently grew *Pseudomonas aeruginosa*, there were no systemic or local features of invasive infection after debridement. The isolate was therefore interpreted as colonization, and management remained directed toward wound-bed optimization and perfusion rather than systemic antipseudomonal therapy.

**Figure 3:**
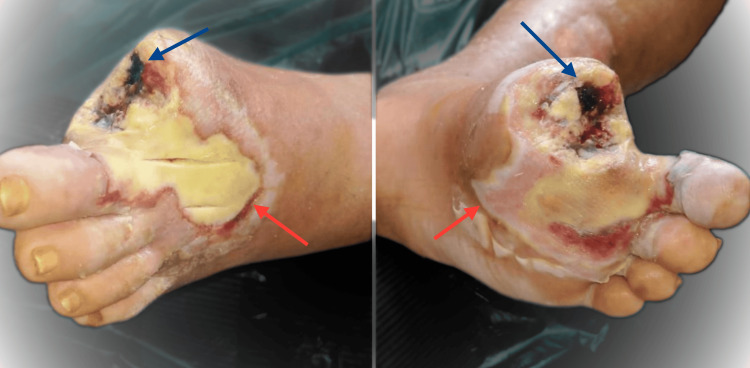
Following amputation of the left great toe, a necrotic patch over the wound can be seen with small areas of bleeding (blue arrows). The surrounding area is discolored with a line of demarcation around the affected area (red arrows).

Despite these measures, ischemia progressed, with a necrotic, discharging lesion developing over the plantar aspect of the left great toe. The patient underwent amputation of the great toe (Figure 3) following failure of conservative wound care.

**Figure 4:**
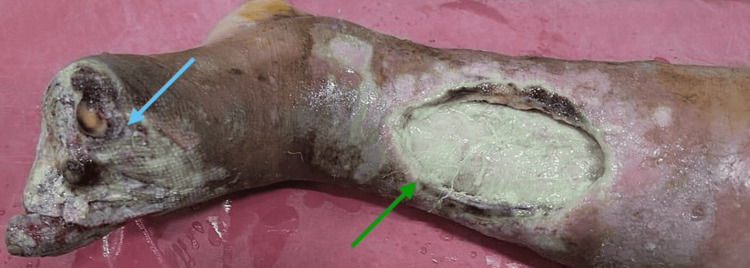
Post-amputation image of the left forefoot. The amputated wound showed signs of infection and decay with constant discharge and was non-healing (light blue arrow). The leg also had a large, open, deep ulcerative wound on the medial surface above the ankle that was infected and the skin around the ulcer discolored, with areas of necrosis and a buildup of slough and exudate (green arrow).

Within one month, the disease process advanced despite prior intervention, culminating in a forefoot amputation (Figure 4) for progressive, non-healing ulceration with tissue necrosis. Intra-operative cultures were not obtained; however, chronic osteomyelitis was considered, given the longstanding course and impaired wound recovery.

Following these complications, the patient was referred to Rheumatology for further evaluation. Autoantibody testing revealed antinuclear antibody (ANA) positivity (+++, 1:1000, centromere pattern), ANA blot positive for CENP-B, anti-Sm antibody positivity, and anti-U1-RNP antibody positivity, establishing a diagnosis of systemic sclerosis-mixed connective tissue disease.

Given her dyspnea and the risk of pulmonary arterial hypertension (PAH) in connective tissue disorders, transthoracic echocardiography demonstrated preserved left ventricular ejection fraction (60%), no regional wall motion abnormalities, and no pulmonary artery hypertension, thereby excluding cardiac dysfunction or PAH as contributors to breathlessness at that time. This pattern is consistent with the literature identifying microcirculatory failure as the principal driver of ischemia in systemic sclerosis, rather than cardiac or pulmonary causes [13].

To clarify the nature of the wound bed and the basis for poor recovery at the amputation site, representative tissue from the stump was submitted for histopathological examination. Sections showed fibroadipose tissue with fibrocollagenous proliferation, dense acute inflammatory infiltrates with necrosis, thrombosed vessels, and mural calcifications, findings in keeping with acute suppurative inflammation, not suggestive of primary vasculitis.

Evaluation of the arterial tree was undertaken with CT angiography to delineate the extent and distribution of disease for potential limb-sparing intervention. The study demonstrated diffuse atherosclerotic change with focal mid-peroneal narrowing and reduced flow, severe anterior tibial stenosis with absent distal opacification, and mild diffuse narrowing of the posterior tibial artery.

Given these anatomical constraints and the absence of PAH, tadalafil and prazosin were withdrawn. Management was redirected toward optimization of the wound milieu, including debridement, dressings, and broad-spectrum coverage for mixed flora, together with vasodilation for ischemic microvascular disease (nifedipine) and immunomodulation (hydroxychloroquine). The patient was re-referred to Plastic Surgery to consider advanced reconstructive strategies, including microvascular approaches, where feasible.

**Figure 5:**
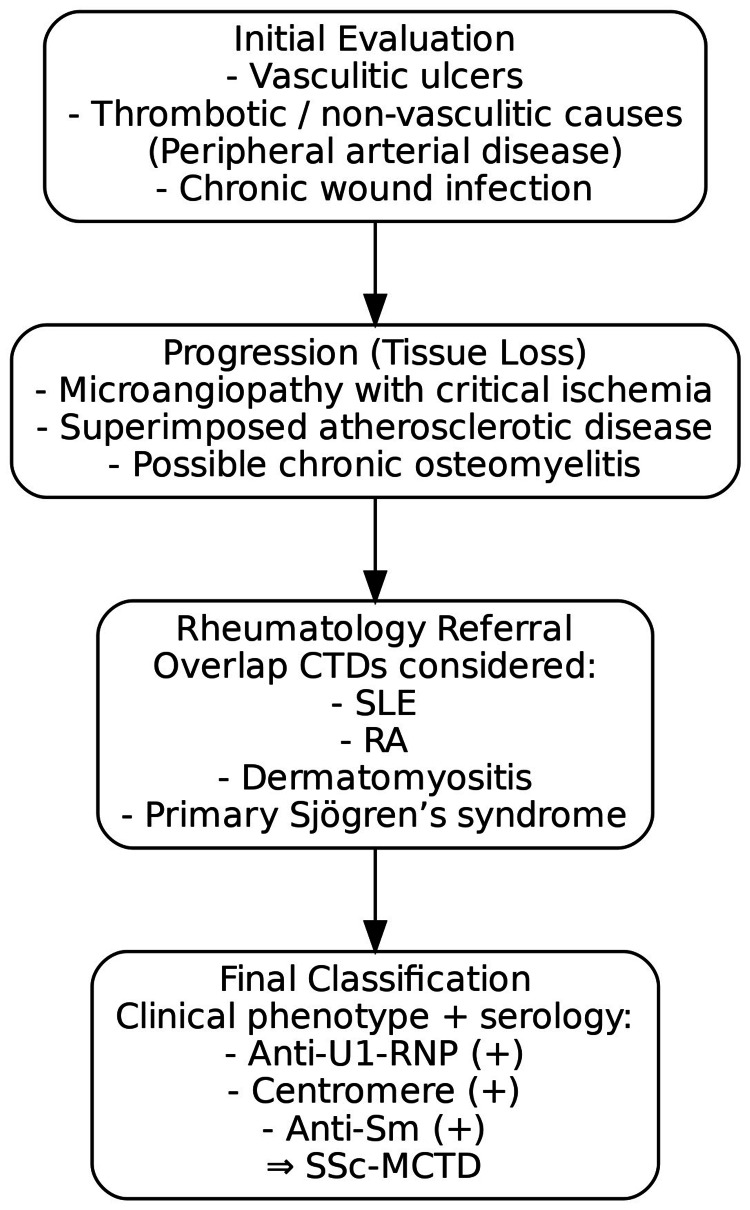
Stepwise diagnostic considerations from initial ulcer evaluation to the final classification of systemic sclerosis–mixed connective tissue disease (SSc-MCTD) SLE, systemic lupus erythematosus; RA, rheumatoid arthritis

To contextualize the diagnostic reasoning, the progressive narrowing of differentials at each stage, from vascular and infective causes to overlap connective tissue diseases, leading to the final classification of SSc-MCTD is illustrated in Figure 5.

## Discussion

Pathogenesis and principles of medical management

Management of non-healing digital ulcers in systemic sclerosis is informed by their pathogenesis [14]. Hallmark vascular changes, such as intimal hyperplasia, adventitial fibrosis, and luminal narrowing, compounded by autoantibody-mediated platelet activation, aggregation, and thrombosis, drive progression from Raynaud’s phenomenon to critical ischemia with ulceration and tissue loss [15,16]. Initial therapy includes calcium-channel blockers, phosphodiesterase-5 inhibitors, endothelin-receptor antagonists, and prostacyclin analogues, combined with non-pharmacological measures such as cold avoidance and smoking cessation. Procedural interventions are considered for ulcers that are severe, recurrent, or refractory to medical management [17]. These include digital sympathectomy, selected revascularization, and early debridement of necrotic tissue to restore a viable wound bed, whereas amputation remains a last resort for established gangrene or infection [17-19]. In some patients, combined pharmacologic therapy and wound care achieve ulcer healing and prevent surgical escalation [20].

Local care, vascular evaluation, and surgical input

In this case, care integrated wound-bed optimization (sharp debridement, dressings, bioburden control) with vascular evaluation to establish the level and severity of arterial disease, which informed decisions on surgical management. At this centre, General Surgery undertakes vascular evaluation and procedures, supported by Plastic Surgery for wound care. Input from a dedicated vascular surgeon, had it been available, could have provided additional nuance in a case of this complexity. This multidisciplinary pathway aligns with the World Scleroderma Foundation recommendations, which advocate local wound optimization combined with systemic measures and surgical collaboration when required [21]. Imaging with Doppler and CT angiography was central in distinguishing microvascular dysfunction from proximal stenosis/occlusion, findings that shaped surgical decision-making. The prognostic implications are significant: macrovascular lower limb ulcers in systemic sclerosis are associated with a high risk of tissue loss, with amputation required in over half of patients in a multicentre study despite vasodilators and revascularization [9,22]. Additional data identify predictors of amputation [23] and highlight the persistent morbidity of lower limb ulcers in systemic sclerosis [24]. These findings reinforce the need for early, coordinated input across specialties to prevent progression to irreversible ischemic injury.

Infection, colonization, and osteomyelitis

Chronic ulcers frequently harbour bacterial growth, and careful distinction between colonization and invasive infection is essential. Moreover, impaired perfusion and immune dysfunction can mimic infection. In this case, *Pseudomonas aeruginosa* isolated from a wound swab after debridement was interpreted as colonization, given the absence of systemic or local features of infection. Wound-bed optimization and perfusion support were therefore prioritized rather than systemic anti-pseudomonal therapy. When infection is clinically apparent, culture-directed antibiotics are indicated, and in chronic or refractory cases, the possibility of osteomyelitis should be investigated [21].

Limb-sparing and reconstruction

Where tissue perfusion is preserved or restored, reconstructive approaches can enable salvage. Autologous skin grafting has been reported as an effective adjunct for severe leg ulcers in systemic sclerosis [25], demonstrating that attention to both perfusion and the wound bed may avert progression to amputation.

Differential diagnoses and justification for SSc-MCTD

The differential diagnoses included vasculitic ulcers, thrombotic occlusion, chronic infection, peripheral arterial disease, and other connective-tissue disease mimics. Distinction relies on a combination of clinical patterning, serological markers, and, when available, histopathology [26]. In this case, the presence of anti-U1-RNP, anti-Sm, and centromere (CENP-B) positivity, together with the clinical phenotype, supported a diagnosis of SSc-MCTD. Series from India and elsewhere confirm that digital gangrene may occur in connective-tissue disorders, with MCTD among leading causes, although progression to major limb amputation remains rare [27].

Blood-pressure strategy and organ surveillance

Comprehensive organ surveillance is essential in systemic sclerosis. Echocardiography in this case excluded pulmonary arterial hypertension and left-ventricular dysfunction, while laboratory studies suggested mild renal involvement, warranting ongoing monitoring. Blood-pressure management requires careful individualization: alpha-blockers may reduce vasospasm but also lower systemic pressure, risking compromised distal perfusion in critical ischemia. Calcium-channel blockade was therefore preferred, reflecting current best practice in this setting.

Limitations

This single-case report limits generalizability. Histopathological confirmation of osteomyelitis was not obtained, and specialist vascular-surgery consultation was unavailable, which may have influenced management. Long-term follow-up data, including post-amputation functional recovery and ulcer recurrence or macrovascular progression, were not available. As a result, our findings are limited to the period of immediate wound healing. These findings are primarily hypotheses, generating and warranting confirmation in larger series.

## Conclusions

Digital ulcers are a clinically meaningful marker of morbidity in systemic sclerosis. This case illustrates how delayed recognition of SSc-MCTD with advancing ischemia can culminate in limb loss, underscoring the need for early, stepwise escalation, including wound-bed optimization, targeted vasodilatory therapy, and timely surgical input within the multidisciplinary team (MDT) pathway. Systemic sclerosis should not be pursued in every ulcer, but should be considered when suggestive features are present (e.g., Raynaud’s phenomenon, characteristic skin changes, supportive serology). Antimicrobial therapy is reserved for clinically evident infection, and antihypertensives should be tailored to preserve distal perfusion. Amputation should be reserved as a last resort when tissue viability is irreversibly lost.
